# Improved Bat Algorithm Applied to Multilevel Image Thresholding

**DOI:** 10.1155/2014/176718

**Published:** 2014-08-03

**Authors:** Adis Alihodzic, Milan Tuba

**Affiliations:** ^1^Faculty of Mathematics, University of Sarajevo, 71000 Sarajevo, Bosnia And Herzegovina; ^2^Faculty of Computer Science, Megatrend University Belgrade, 11070 Belgrade, Serbia

## Abstract

Multilevel image thresholding is a very important image processing technique that is used as a basis for image segmentation and further higher level processing. However, the required computational time for exhaustive search grows exponentially with the number of desired thresholds. Swarm intelligence metaheuristics are well known as successful and efficient optimization methods for intractable problems. In this paper, we adjusted one of the latest swarm intelligence algorithms, the bat algorithm, for the multilevel image thresholding problem. The results of testing on standard benchmark images show that the bat algorithm is comparable with other state-of-the-art algorithms. We improved standard bat algorithm, where our modifications add some elements from the differential evolution and from the artificial bee colony algorithm. Our new proposed improved bat algorithm proved to be better than five other state-of-the-art algorithms, improving quality of results in all cases and significantly improving convergence speed.

## 1. Introduction

Image segmentation is process of subdivision of an image into homogeneous and disjoint sets sharing similar properties such as intensity, color, and contours. Homogeneous sets are introduced with respect to a certain criterion of homogeneity. Image segmentation usually represents the first step in image understanding and representation and the results obtained by segmentation are used for further high-level methods such as feature extraction, semantic interpretation, image recognition, and classification of objects. In general, image segmentation simplifies the process of dividing an image into regions that are used for further specific applications. Several practical applications cover character recognition [1], detection of video changes [2], medical imaging [3, 4], automatic target recognition [5], and so forth. Over the last few decades a lot of algorithms for image segmentation, either for gray level or color images, were presented in the literature. Good review of these algorithms can be found in [6]. In general, image segmentation algorithms can be grouped into thresholding, edge-based, region-grow, and clustering.

Image thresholding is one of the most widespread segmentation techniques that performs image segmentation based on the information contained in the global gray value of the image histogram. Thresholding is called bilevel thresholding in the case that an image is separated into two classes, one including those pixels with gray levels above a specified threshold and the other including the rest. Unlike bilevel thresholding, multilevel thresholding performs subdivision of an image into several classes. In this case the pixels belonging to the same class take gray levels from the intervals defined by successive thresholds. Multiple pixels belonging to the same class are not always homogeneous and may be represented by different feature values. Selection or computing of the multilevel thresholds is crucial in image segmentation since proper segmentation depends on adequately computed thresholds.

There are many different methods for computing the thresholds for an image such as maximizing the gray level variance [7], entropy [8], similarity [9], and measure of fuzziness [10]. In general, thresholding methods can be divided into parametric and nonparametric methods. Using parametric methods, such as a novel image thresholding method based on Parzen window estimate [11], nonsupervised image segmentation based on multiobjective optimization [12], a multilevel thresholding approach using a hybrid optimal estimation algorithm [13], and optimal multithresholding using a hybrid optimization approach [14], may involve the solution of nonlinear equations which increases of the computational complexity. Therefore, the nonparametric methods [15] are introduced for finding the thresholds by optimizing some discriminating criteria. Among the mentioned different thresholding criteria, the entropy is the most popular optimization method. Using the entropy of the histogram, Pun was the first to introduce a new method for gray level image thresholding [8]. Later, this method was corrected and improved by Kapur et al. [16], since Kapur found some artifacts in Pun's method. Sahoo used Shanon's concept of entropy, considering two probability distributions for background and foreground objects. He has proposed a thresholding technique based on Renyi's entropy [17]. Information about the gray value of each pixel and the average value of its immediate neighborhood are obtained by two-dimensional entropy which is calculated by two-dimensional histogram.

Another important group of methods based on discriminant analysis is the clustering-based methods [18]. In these methods, gray values are clustered into several classes, so that there is a similarity of gray values within the class and dissimilarity between classes. To perform the separation of classes, Otsu has developed a thresholding method for computing the optimal thresholds by maximizing the between-class variance using an exhaustive search [7]. It has been shown that this method gives acceptable results when the number of pixels in each class is close to each other. For bilevel image thresholding, the above-mentioned methods are effective. However, for the optimal multilevel thresholding, the existing conventional methods are being hindered by an exhaustive search when the number of thresholds is increased. To overcome this problem, powerful metaheuristics are used to search for the optimal thresholds in order to achieve a fast convergence and reduce the computational time.

Metaheuristics are optimization methods that orchestrate an interaction between local improvement procedures and higher level strategies to create a process capable of escaping from local optima and performing a robust search of a solution space [19, 20]. Several metaheuristic algorithms derived from the behavior of biological and physical systems in the nature have been proposed as powerful methods for searching the multilevel image thresholds. Since magic algorithm that works for all problems does not exist [21], different approaches have been developed for different classes of problems such as combinatorial or continuous, with additions for constrained optimization problems [22]. Original versions of metaheuristic algorithms are often modified or hybridized in order to improve performance on some classes of problems. The most popular nature-inspired algorithms for optimization, with improvements, adjustments, and hybridizations, include particle swarm optimization (PSO) [23], differential evolution (DE) [24], firefly algorithm (FA) [25, 26], cuckoo search (CS) [27–29], ant colony optimization [30–33], artificial bee colony algorithm [34–38], bat algorithm (BA) [39, 40], and human seeker optimization (HSO) [41–43].

DE algorithm has been adapted for searching the optimal multilevel thresholds [44]. PSO algorithm modified by Yin to search for the thresholds can be found in [45]. Akay presented a comprehensive comparative study of the ABC and PSO algorithms for finding multilevel thresholds using Kapur's and Otsu's criteria [46]. Maitra and Chatterjee proposed an improved variant of PSO algorithm for the task of image multilevel thresholding [47]. The results showed that the ABC algorithm with both the between-class variance and the entropy criterion can be efficiently used in multilevel thresholding. Hammouche focused on solving the image thresholding problem by combining between-class variance criterion with metaheuristic techniques such as GA, PSO, DE, ACO, SA, and TS [48].

In this paper, we adapted the bat algorithm for multilevel image thresholding. Bat algorithm is simple to implement and produces good results. However, based on our experiments, it is powerful in intensification, but at times it may get trapped into local optima when it is applied to some difficult problems. Therefore, we propose an improved version of bat algorithm adopted to search for multilevel thresholds using Kapur and Otsu criteria. Our proposed modification merges three approaches to produce a new improved bat-inspired (IBA) algorithm according to the principle of bat algorithm, differential evolution, and some scout technique taken from the ABC algorithm. We compared our proposed algorithm with state-of-the-art algorithms from [49]. The experimental results show that the proposed IBA algorithm always gives better results compared to PSO, DE, CS, FA, and BA algorithms, considering both accuracy and, especially, convergence speed.

The remainder of the paper is organized as follows.  Section 2 describes the multilevel thresholding problem and presents Kapur's and Otsu's objective functions.  Section 3 and Section 4 describe the original BA and IBA algorithms adopted to search for the optimal multilevel thresholds, respectively.  Section 5 shows the experimental and comparative results of applying PSO, DE, CS, FA, BA, and IBA to multilevel segmentation to standard benchmark images. Finally, our conclusions are discussed in Section 6.

## 2. Multilevel Image Thresholding

Thresholding technique performs image segmentation based on the information contained in the image histogram. If we consider a gray-scale input image *I* as a set of pixels *A*, multilevel thresholding can be defined as a method of dividing the set *A* into *n* + 1 disjoint subsets (*A*_0_, *A*_1_,…, *A*_*n*_) by some numbers (*α*_0_, *α*_1_,…, *α*_*n*−1_) such that

(1) A0={x:0≤f(x)<α0},A1={x:α0≤f(x)<α1},⋮An={x:αn−1≤f(x)≤L−1},

where *x* = (*x*_1_, *x*_2_) is a pixel defined by coordinates *x*_1_ and *x*_2_ in the Cartesian coordinate system, *f*(*x*) presents a gray level value of pixel *x*, and the *f*(*x*) takes values in the range [0,255]. The aim of multilevel thresholding is to compute the optimal threshold values (*α*_0_, *α*_1_,…, *α*_*n*−1_). The sets (*A*_0_, *A*_1_,…, *A*_*n*_) may represent different regions of the object. It is clear that *A*_*i*_  ∩  *A*_*j*_ = *ø*, and their union presents the whole input image *I*.

Optimal threshold selection for bilevel thresholding is not computationally expensive, while for multilevel thresholding, computing more than few optimal threshold values is an expensive and time consuming operation. The optimal threshold values can be determined by optimizing some criterion functions defined from the histogram of image. In this paper, we use two popular threshold criteria: Kapur's entropy criterion and Otsu's between-class variance criterion.

### 2.1. Kapur's Thresholding Method

Entropy is a measure of uncertainty proposed by Shannon [50], later widely used [51]. Let *x* be a discrete random variable taking values *x*_*i*_ with probabilities *p*_*i*_, *i* = 1,2,…, *n*, respectively. Then its entropy is defined by

(2) $$\begin{array}{c} H\left(x\right)=-\overset{n}{\underset{i=1}{\sum }}p_{i}\ln\left(p_{i}\right). \end{array}$$

The Kapur's method [16] based on the entropy is used to perform multilevel thresholding. For this method, the threshold criteria can be formulated as follows. Assume that an image *I* contains *n* pixels with gray levels belonging to the set {0,1,…, *L* − 1}. Let *h*(*i*) present the number of pixels at gray level *i*, and *p*_*i*_ = *h*(*i*)/*n* is the probability of occurrences of gray level *i* in the image *I*. The subdivision of an image into *k* + 1 classes can be considered as a *k*-dimensional optimization problem for the calculation of *k* optimal thresholds (*t*_0_, *t*_1_,…, *t*_*k*−1_). The optimal thresholds are obtained by maximizing the objective function:

(3) $$\begin{array}{c} f\left(t_{0},t_{1},\ldots ,t_{k-1}\right)=\overset{k}{\underset{i=0}{\sum }}H_{i}, \end{array}$$

where the entropies *H*_*i*_ are defined by

(4) H0=−∑i=0t0−1piw0ln⁡piw0, w0=∑i=0t0−1pi,H1=−∑i=t0t1−1piw1ln⁡piw1, w1=∑i=t0t1−1pi,⋮Hk=−∑i=tk−1  L−1piwkln⁡piwk, wk=∑i=tk−1L−1pi.

### 2.2. Otsu's Thresholding Method

Otsu's method [7] based on the maximization of the between-class variance is one of the most popular methods proposed for image thresholding. The algorithm for this method can be described as follows. Assume that an image *I* can be represented by *L* gray levels. The probabilities of pixels at level *i* are denoted by *p*_*i*_ so *p*_*i*_ ≥ 0 and *p*_0_ + *p*_1_ + ⋯+*p*_*L*−1_ = 1. Cumulative probabilities for classes *A*_*i*_, *i* = 0,1,…, *k*, can be defined as

(5) $$\begin{array}{c} w_{0}=\overset{t_{0}-1}{\underset{i=0}{\sum }}p_{i},\;\;w_{1}=\overset{t_{1}-1}{\underset{i=t_{0}}{\sum }}p_{i},\ldots ,w_{k}=\overset{L-1}{\underset{i=t_{k-1}}{\sum }}p_{i}, \end{array}$$

where *t*_*j*_ are the thresholds separating these classes. For *k* + 1 classes *A*_*i*_, (*i* = 0,1,…, *k*), the goal is to maximize the objective function:

(6) $$\begin{array}{c} f\left(t_{0},t_{1},\ldots ,t_{k-1}\right)=\overset{k}{\underset{i=0}{\sum }}\sigma_{i}, \end{array}$$

where the sigma functions are defined by

(7) σ0=w0(∑i=0t0−1ipiw0−∑i=0L−1ipi)2,σ1=w1(∑i=t0t1−1ipiw1−∑i=0L−1ipi)2,⋮σk=wk(∑i=tk−1L−1ipiwk−∑i=0L−1ipi)2.

## 3. Bat Algorithm Adapted for Multilevel Image Thresholding

Bat algorithm is a recent metaheuristic introduced by Yang [39], based on so-called echolocation of the bats. In this algorithm, bats detect prey and avoid the obstacles by using the echolocation. Bat algorithm was successfully applied to a number of very different problems like large-scale optimization problems [52], global engineering optimization [53], fuzzy clustering [54], parameter estimation in dynamic biological systems [55], multiobjective optimization [56], image matching [57], economic load and emission dispatch problems [58], data mining [59], scheduling problems [60], neural networks [61], and phishing website detection [62].

In the bat algorithm, bats navigate by using time delay from emission to the reflection. The pulse rate can be simply determined in the range from 0 to 1, where 0 means that there is no emission and 1 means that the bat's emitting is at maximum. Apart from the control parameters, such as the population size and maximum iteration number which are common control parameters for all nature inspired algorithms, the BA has few important parameters such as frequency tuning parameter similar to the key feature used in the PSO and HS, parameter for automatically zooming into a region where the promising solutions have been found, and the control parameter for automatically switching from exploration to exploitation. This gives advantage to the BA over other metaheuristic algorithms in the literature.

In order to implement the bat algorithm, the following three idealized rules are used [39]:

 (i) all bats use echolocation to sense distance, and they also “know” the surroundings in some magical way;
 (ii) bats fly randomly with velocity *v*_*i*_ at position *x*_*i*_ with a fixed frequency *f*_min⁡_, varying wavelength *λ*, and loudness *A*_0_ to search for prey. They can automatically adjust the wavelength of their emitted pulses and adjust the rate of pulse emission *r* from [0,1], depending on the proximity of their target;
(iii)  although the loudness can vary in many ways, it is assumed that the loudness varies from a positive large value *A*_0_ to a minimum constant value *A*_min⁡_.

The proposed bat algorithm tries to select *k* threshold values which maximize the fitness functions which are described by (3) and (6), respectively. The details of the developed BA approach for multilevel image thresholding are given as follows.

*Step 1 (generate initial population of solutions)*. The bat algorithm generates a randomly distributed initial population of *N* solutions (bats) (*i* = 1,2,…, *N*), where each solution has *k* dimensions. All solutions can be presented by matrix *X*:

(8) $$\begin{array}{c} X=\left[\begin{array}{ccccc} x_{1,1} & x_{1,2} & x_{1,3} & \cdots & x_{1,k}\\ x_{2,1} & x_{2,2} & x_{2,3} & \cdots & x_{2,k}\\ & & & & \vdots \\ x_{N,1} & x_{N,2} & x_{N,3} & \cdots & x_{N,k} \end{array}\right], \end{array}$$

where *x*_*i*,*j*_ is the *j*th component value that is restricted to {0,…, *L* − 1} and *x*_*i*,*j*_ < *x*_*i*,*j*+1_ for all *j*. The fitness values for all solutions are evaluated and variable *cycle* is set to one. The bat algorithm detects the most successful solution as *x*_best_ before starting iterative search process.

*Step 2 (calculation of new solutions)*. Calculation of a new solution *x*_*i*_^*t*^ is performed by moving virtual bats *x*_*i*_^*t*−1^ according to equation

(9) $$\begin{array}{c} x_{i}^{t}=x_{i}^{t-1}+v_{i}^{t}, \end{array}$$

where *v*_*i*_^*t*^ denotes the bat velocity of movement, and it is calculated by formula

(10) $$\begin{array}{c} v_{i}^{t}=v_{i}^{t-1}+\left(x_{i}^{t}-x_{\text{best}}\right)*f_{i}. \end{array}$$

In (10), *f*_*i*_ denotes the frequency and *x*_best_ denotes the current global best solution. The frequency *f*_*i*_ can be calculated as

(11) $$\begin{array}{c} {f_{i}^{t}}^{\;}=f_{\min}+\left(f_{\max}-f_{\min}\right)*\beta , \end{array}$$

where *β* is a random vector generated by a uniform distribution belonging to the closed interval [0,1]. For min and max frequency, the recommended values *f*_min⁡_ = 0 and *f*_max⁡_ = 2 are used. In this computation step, the bat algorithm controls the boundary conditions of the calculated new solution *x*_*i*_^*t*^. In the case that the value of a variable overflows the allowed search space limits, then the value of the related variable is updated with the value of the closer limit value to the related variable.

*Step 3 (improving the current best solution)*. For each solution *x*_*i*_^*t*^ apply the next operator which is defined by

(12) $$\begin{array}{c} x_{\text{new}}=\{\begin{array}{cc} x_{\text{best}}+\epsilon A_{t}, & \text{if }\;\;\;rand_{1}>r_{i}^{t},\\ x_{i}^{t}, & \text{otherwise}, \end{array} \end{array}$$

where *rand*_1_ is a uniform random number in range [0,1], *ϵ* is a scaling factor drawn from uniform distribution in the range [−1,1], *A*_*t*_ = 〈*A*_*i*_^*t*^〉 is the average loudness of all bats at the computation step *t*, and *r*_*i*_^*t*^ is the pulse rate function. The pulse rate function is defined by

(13) $$\begin{array}{c} r_{i}^{t}=r_{i}^{0}\left(1-e^{-\beta t}\right), \end{array}$$

where *β* is a constant and *r*_*i*_^0^ are initial pulse rates in the range [0,1]. It can be seen from (12) that this function controls the intensive local search depending on the value of uniform variable *rand*_1_ and the rate *r*_*i*_^*t*^. Also, at this step, the BA controls the boundary conditions at each iteration.

*Step 4 (acceptation of a new solution by flying randomly)*. In this step, the solution *x*_new_ obtained in Step 3 is accepted as a new solution and *f*(*x*_new_) as a new objective function value by using

(14) (xit,fit(xit))={(xnew,f(xnewt)),if(rand2<Ait  and f(xnewt)>f(xit−1)),(xit−1,f(xit−1)),  otherwise,

where *rand*_2_ is a uniform random number in range [0,1] and *A*_*i*_^*t*^ is the loudness function defined by

(15) $$\begin{array}{c} A_{i}^{t}=\alpha A_{i}^{t-1}, \end{array}$$

where *α* is a constant and plays a similar role as the cooling factor of a cooling schedule in the simulated annealing. Therefore, if the solution *x*_new_ has the higher objective function value compared to the old solution *x*_*i*_^*t*−1^ and the loudness *A*_*i*_^*t*^ is more than *rand*_2_, then the new solution is accepted, the old fitness value is updated, and functions defined by (13) and (15) are updated, too. Otherwise, the new solution *x*_new_ is abandoned, and the old best solution is kept.

*Step 5 (memorize the best current solution)*. Record the best solution so far (*x*_best_), that is, the solution with the highest objective function value.

*Step 6 (check the stopping criteria)*. If the termination criterion is met or the variable *cycle* is equal to the maximum number of iterations, then the algorithm is finished. Otherwise, increase the variable *cycle* by one and go to Step 2.

## 4. Our Proposed Improved Bat Algorithm: IBA

As described in the previous section, we selected the BA for multilevel image thresholding. BA is simple to implement and it produces good results when the number of thresholds is small. However, based on our experiments, the BA algorithm often fails when the number of thresholds is larger, especially for the Kapur's objective function. Therefore, an adjustment of the bat algorithm was required. In this paper, the improved hybridized bat algorithm (IBA) is proposed to overcome the mentioned drawback of the pure bat algorithm. It combines two different solution search equations of the bat algorithm and DE algorithm [24]. The IBA algorithm includes differential operators mutation and crossover from DE algorithm, with the aim of speeding up convergence and to achieve a good balance between intensification and diversification. Mutation and crossover operators are used to improve the original BA generation of a new solution for each bat so that the IBA can more efficiently explore and exploit the new search space and avoid being trapped into local optima.

In the pure BA, exploration and exploitation are controlled by pulse rate function (13). Analyzing this function we noticed that the algorithm loses exploration capability as iterations progress. The form of this function makes switching from the exploration to exploitation and vice versa possible. In this way, the exploration capability of BA can be modified by inserting differential operators for crossover and mutation [24] instead of (12) and for the exploitation capability (12) continues to be used for a good intensification. Therefore, a good balance is established between intensification and diversification.

Although the above modification can improve many solutions, some solutions will still remain stuck in some local optimum. In order to fix this lack of the former modification, we introduced the second modification which is inspired by launch of the scouts in the scout phase of the ABC algorithm. When some solution gets trapped in a local optimum after a certain number of iterations, it will eventually exceed the predetermined number of allowed trials called “limit.” When a solution exceeds the “limit” trials unchanged, it is redirected to search new space by using the random walk.

In the proposed IBA algorithm, bats form a population of threshold values. The threshold values produced by the bat *i* are noted as *x*_*i*_ = (*x*_*i*,1_, *x*_*i*,2_,…, *x*_*i*,*k*_), *i* = 1,…, *N*. All bats perform searching in the solution search space with the aim to optimize the objective functions described by (3) or (6). The details of the proposed IBA approach for multilevel thresholding are given as follows.

*Step 1 (generate the initial population of solutions)*. The IBA begins by randomly generating population with *k* dimensions as in the case of the proposed BA approach for multilevel thresholding. Each threshold value *x*_*i*,*j*_(*i* = 1,…, *n*; *j* = 1,…, *k*) of the matrix *X* generated by the bat *i* is restricted to set {0,1,…, *L* − 1} and for all *j* holds *x*_*i*,*j*_ < *x*_*i*,*j*+1_. Also, at this step initialization is done for the parameter* limit* which presents the number of allowed attempts to improve a bat, the initial loudness *A*_*i*_ and pulse rate *r*_*i*_^0^, as well as the initial values of the parameters in the DE algorithm such as the differential weight *F* and crossover probability *C*_*r*_. After generation of the initial population, the fitness value for each solution *x*_*i*_ is evaluated. Then the IBA algorithm detects the most successful solution as *x*_best_, before starting iterative search process. After that it sets the variable *cycle* to one.

*Step 2 (calculate the new population)*. Calculation of a new threshold *x*_*i*_^*t*^ is performed by moving virtual bats *x*_*i*_^*t*−1^ according to (9). The velocity *v*_*i*_^*t*^ and frequency *f*_*i*_ are calculated by (10) and (11), respectively. At this computation step, the IBA controls the boundary conditions of the calculated new solution *x*_*i*_^*t*^. In the case that the value of the *x*_*i*_^*t*^ is less than 0 or is more than *L* − 1, then the value of the *x*_*i*_^*t*^ is updated with the value of the closer limit value to the variable *x*_*i*_^*t*^.

*Step 3 (improving the current best solution by differential operators)*. For each solution *x*_*i*_^*t*^ apply the next operator which is defined by

(16) $$\begin{array}{c} x_{\text{new}}=\{\begin{array}{cc} x_{\text{dif}}^{t}, & \text{if}\;\;rand_{1}>r_{i}^{t},\\ x_{\mathrm{loc}}^{t}, & \text{otherwise}, \end{array} \end{array}$$

where *rand*_1_ is randomization term in the range [0,1], *r*_*i*_^*t*^ is the pulse rate function defined by (13), *x*_dif_^*t*^ is the differential operator for mutation and crossover, and *x*_loc⁡_^*t*^ is the operator based on the local search in the BA. The differential mutation and crossover operations are performed by

(17) $$\begin{array}{c} x_{\text{dif},j}^{t}=\{\begin{array}{cc} x_{c,j}^{t}+F\left(x_{a,j}^{t}-x_{b,j}^{t}\right), & \text{if}\;\left(rand_{2}<C_{r}\;\text{or}\;j=j_{r}\right),\\ x_{i,j}^{t}, & \text{otherwise}, \end{array} \end{array}$$

where *x*_*a*_, *x*_*b*_, and *x*_*c*_ are three randomly chosen different vectors in the range [0, *N* − 1] at the cycle *t*, *F* is the differential weight that scales the rate of modification, *C*_*r*_ is the crossover probability in the interval [0,1], *j*_*r*_ is randomly selected in the range [0, *k*], and *rand*_2_ is a uniform variable in the range [0,1]. Inside the implementation of the differential operator *x*_dif_, the boundary conditions for all *j*  (*j* = 1,…, *k*) are controlled. As an important improvement of the proposed method, the binomial “DE/rand/1/bin” scheme is used in order to increase the diversity of the bats and achieve both the precision and search efficiency. The local search is performed by

(18) $$\begin{array}{c} x_{\mathrm{loc},j}^{t}=\{\begin{array}{cc} x_{l\text{best},j}^{t}, & \text{if}\;\left(f\left(x_{l\text{best},j}^{t}\right)>f\left(x_{i,j}^{t}\right)\right),\\ x_{i,j}^{t}, & \text{otherwise}, \end{array} \end{array}$$

where *x*_*l*best,*j*_^*t*^ is defined by

(19) $$\begin{array}{c} x_{l\text{best},j}^{t}=x_{\text{best},j}^{t-1}+\epsilon A_{i,j}^{t-1}. \end{array}$$

As in the ordinary BA, parameters *ϵ* and *A*_*i*,*j*_ denote the scaling factor and the loudness function, respectively. Also, inside the local search operator *x*_loc⁡_, the boundary conditions for all *j*  (*j* = 1,…, *k*) are checked. In our proposed approach, we found that it is beneficial to replace (13) by *r*_*i*_^*t*^ = *r*_*i*_^0^(1 − *β* ^*t*^). It will be shown in experimental study that the best results are obtained for initial pulse rates *r*_*i*_^0^ = 0.5, initial loudness *A*_0_ = 0.95, and *β* = 0.9.

*Step 4 (acceptation of a new solution by flying randomly)*. In this step, the solution *x*_new_ obtained in Step 3 is accepted as a new solution and *f*(*x*_new_) as a new objective function value by using

(20) (xit,fit(xit))={(xnewt,f(xnewt)), if(rand3<Ait  and f(xnewt)>f(xit−1)),(xit−1,f(xit−1)) tri=tri+1, otherwise,

where *rand*_3_ is a random number in the range [0,1], *tr*_*i*_ is a vector recording the number of attempts through which solution *x*_*i*_^*t*^ could not be improved at cycle *t*, and *A*_*i*_^*t*^ is defined by (15). In the above equation, if the solution *x*_*i*_^*t*−1^ cannot be improved, then the new solution *x*_new_ is abandoned and the *i*th element of the trial vector tr is increased by one. Also, after certain number of cycles determined by the variable limit, if the solution *x*_*i*_^*t*^ cannot be further improved, it is abandoned and replaced by randomly generated solution. In this case, the *i*th element of the trial vector is set to 0. This modification can improve the exploration process and it will help to avoid trapping into some local optima. Also, it will improve the solution quality and speed convergence.

*Step 5 (memorize the best current solution)*. Record the best solution so far (*x*_best_), that is, the solution with the highest objective function value.

*Step 6 (check the stopping criteria)*. If the termination criterion is met or the variable *cycle* is equal to the maximum number of iterations, then the algorithm is finished. Otherwise, increase the variable *cycle* by one and go to Step 2.

## 5. Experimental Results

The multilevel image thresholding problem deals with finding optimal thresholds within the range [0, *L* − 1] that maximize the functions defined by (3) and (6). The dimension of the optimization problem is the number of thresholds *k*, and the search space is [0, *L* − 1]^*k*^. In this study our proposed IBA algorithm was compared against four other standard population based metaheuristic techniques: PSO, DE, CS, and FA from [49] and pure BA.

**Figure 1:**
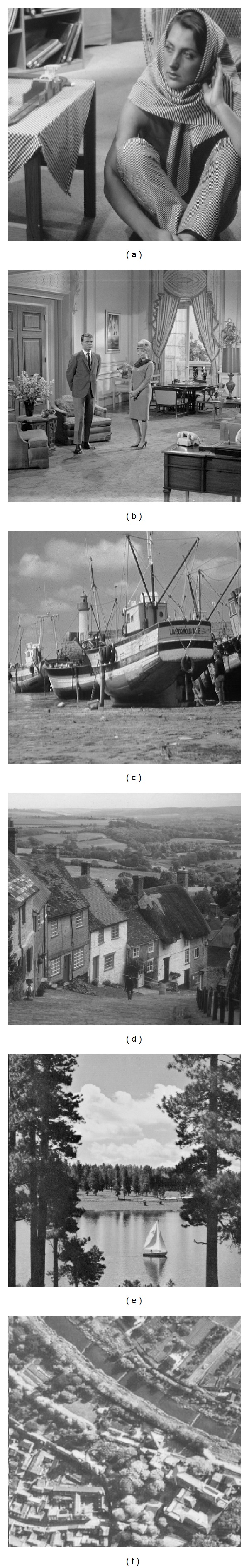
Test images: (a) Barbara, (b) Living room, (c) Boats, (d) Goldhill, (e) Lake, and (f) Aerial.

**Figure 2:**
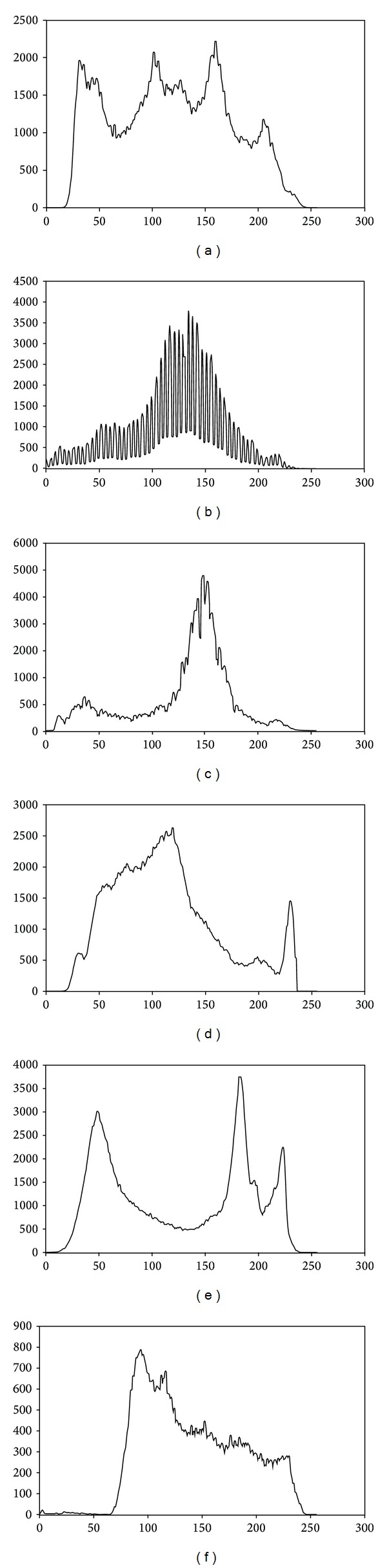
Gray-level histogram of test images: (a) Barbara, (b) Living room, (c) Boats, (d) Goldhill, (e) Lake, and (f) Aerial.

The experiments were conducted on 6 standard images, the same as used in [49], in order to make comparison of the obtained results simpler. Images used in this paper, namely, Barbara, Living room, Boats, Goldhill, and Lake, are of size (512 × 512) and Aerial has size (256 × 256). Barbara and Boats images are available at http://decsai.ugr.es/~javier/denoise/test_images/. The Living room and Lake images were chosen from http://www.imageprocessingplace.com/root_files_V3/image_databases.htm. The Goldhill image can be found at https://ece.uwaterloo.ca/~z70wang/research/quality_index/demo.html. The Aerial image was taken from the University of Southern California Signal and Image Processing Institute's image database at http://sipi.usc.edu/database/database.php?volume=misc. These original images and their gray level histograms are depicted in Figures 1 and 2, respectively.

For the Kapur's and Otsu's thresholding methods, the exhaustive search method was conducted first to derive the optimal solutions, the corresponding optimal objective function values, and the processing time for comparison with the results generated by the PSO, DE, CS, FA, BA, and IBA algorithms. These results generated by the exhaustive search for Kapur's and Otsu's criterion are presented in Tables 1 and 2, respectively. It is obvious that computational times increase exponentially and for more than 5 thresholds become unacceptable. We did not implement optimal use of multicore processor, but improvements would not be significant.

The number of thresholds *k* explored in the experiments were 2, 3, 4, and 5. Since metaheuristic algorithms have stochastic characteristics, each experiment was repeated 50 times for each image and for each *k* value. Each run of an algorithm was terminated when the fitness value of the best solution *f*(*x*_best_) reached the known optimal value (from the exhaustive search) of the objective function *f*_opt_, that is, |*f*(*x*_best_) − *f*_opt_| < *ϵ*, where *ϵ* = 10^−9^ was a tolerance for the accuracy of the measurement. Hence, the stopping condition for all algorithms was the value of the fitness, unless optimum could not be reached within 2000 iterations.

The proposed IBA method has been implemented in C# programming language, as the rest of the algorithms. Results for CS and FA are from [49]. All tests were done on an Intel Core i7-3770K @3.5 GHz with 16 GB of RAM running under the Windows 8 x64 operating system. The PSO and DE algorithms have been implemented in their basic versions, while the BA and IBA have been implemented as it was described in the previous two sections.

### 5.1. Parameters Setup

To compare the proposed IBA algorithm with PSO, DE, CS, FA [49], and BA algorithms, the objective function evaluation was computed *N* × *G* times, where *N* is the population size and *G* is the maximum number of generations (unless optimum was reached earlier). The population size in all algorithms was set to *N* = 40 and the number of generation is set to *G* = 2000 for all algorithms, as in [49]. Besides these common control parameters, each of mentioned algorithms has additional control parameters that directly improve their performance. For both the proposed IBA and pure BA algorithms, the additional control parameters *f*_min⁡_ and *f*_max⁡_ were set to 0 and 2.0, respectively. The initial values for parameters *r*_*i*_^0^ and loudness *A*_*i*_ were set to 0.5 and 0.99, respectively. The constant *β* was set to 0.9. Instead of the average loudness 〈*A*_*i*_^*t*^〉 of all bats, we found that the value 1.66 was acceptable for all images. In the proposed IBA algorithm, control parameters introduced from DE algorithm, such as differential weight *F* and crossover probability *C*_*r*_, were set to 0.75 and 0.95, respectively. Also, in the IBA method, the parameter limit was set to 150.

### 5.2. Quality and Computational Analysis of the Results

The mean and standard deviations for 50 runs for six tested metaheuristic algorithms have been calculated and are presented in Table 3 for the experiments based on Kapur's entropy and in Table 4 for the experiments based on Otsu's objective function. These mean values can be compared to the optimal values of the corresponding objective functions found by an exhaustive search from Tables 1 and 2.

The first conclusion that can be drown from the results in Tables 3 and 4 is that the cases when the number of desired thresholds is 2 or 3 are too easy and are not interesting for nondeterministic metaheuristics. Almost all algorithms in almost all cases reached optimal results (PSO and DE had few misses). We included these results in the tables for comparison with results in [49], but we will not discuss them further. All the remaining discussion is only about cases when the number of desired thresholds is 4 or 5.

From Tables 3, 4, 5, and 6 many details can be seen. We will here, in three additional tables, synthetize the most important conclusions concerning the quality of the results and the convergence speed.

Table 7, computed from Tables 3 and 4, shows for each tested algorithm in what percentage of cases it achieved the best result, considering all tested images and both optimization criteria. From Table 7, we can see that PSO and DE were very inferior compared to other tested algorithms. The results for the CS and FA [49] algorithms are quite acceptable, where FA had slightly better results.

For the BA we can notice that it gives rather poor results for the Kapur's method, while it gives rather good results for the Otsu's method. When the Kapur's criterion is used, the BA gets trapped in local optima, so it consumes the maximum number of iterations without switching to another subspace which is more promising. That explains why it needed some modifications to be introduced to help it leave the local optimum space and continue to search new spaces.

Our proposed improved IBA algorithm, by taking some features of the DE and ABC algorithms, obtained the best results compared to the rest of algorithms. It actually achieved the best result for both mean value and variance, for all tested cases.

Tables 5 and 6 report the mean number of iterations and the average CPU time taken by each algorithm to satisfy the stopping condition for Kapur's and Otsu's criteria, respectively. Most significant conclusions concerning the convergence speed of the tested algorithms are shown in Tables 8 and 9.

In Table 8 (for Kapur's criterion) in each column labeled by Thrs.   *k*  (*k* = 2,3, 4,5) we calculated for each of the tested algorithms: PSO, DE, CS, FA, BA, and IBA, the sum of mean number of required iterations for each test image. We can observe that in the case of the FA and especially the IBA method, the number of iterations does not grow rapidly with the increase of the number of thresholds as is the case with the rest of algorithms. From Table 8 we can also observe that the proposed IBA converges in considerably less iterations compared to the rest of algorithms.

From Table 9 (for the Otsu's criterion), it can be seen that the proposed IBA method in this case also converges in considerably less iterations compared to the other methods. It also maintains the feature of linearity with increasing the number of thresholds. Actually, in both cases, for Kapur's and Otsu's criteria, our proposed IBA algorithm improved the convergence speed by more than a factor of 2, compared to the next best algorithm.

## 6. Conclusion

In this paper, we considered an important optimization problem of multilevel image thresholding. It is an exponential problem and as such it is appropriate for swarm intelligence metaheuristics. We adapted new bat algorithm for this problem and compared it to other state-of-the-art algorithms from [49]. Pure version of the bat algorithm performed well, but the results were slightly below the average, especially when Kapur's criterion was used. We determined that the pure bat algorithm, when applied to this problem, may be easily trapped into local optimum so we modified it by changing new solution equation by hybridized one with elements from DE. We also included limit parameter similar to the one used in the ABC algorithm.

Our proposed improved bat-inspired hybridized with DE (IBA) algorithm was tested on 6 standard benchmark images, the same as used in [49]. It proved to be superior to all other tested algorithms considering the quality of the solutions (it actually achieved the best result for both mean value and variance, for all tested cases), especially it significantly improved convergence speed (more than two times better than the next algorithm). This shows that our proposed algorithm is excellent choice for the multilevel image thresholding problem. Additional adjustments can be done in the future using larger set of synthetic images which will allow more precise modifications and parameter adjustment.

## Figures and Tables

**Table 1:** Thresholds, objective function values, and time processing provided by the exhaustive search for Kapur's method.

Images	*K*	Threshold values	Objective function	Time (ms)
Barbara	2	96, 168	12.668336540	25
	3	76, 127, 178	15.747087798	341
	4	60, 99, 141, 185	18.556786861	11103
	5	58, 95, 133, 172, 210	21.245645310	666869

Living room	2	94, 175	12.405985592	31
	3	47, 103, 175	15.552622213	339
	4	47, 98, 149, 197	18.471055578	12612
	5	42, 85, 124, 162, 197	21.150302316	478114

Boats	2	107, 176	12.574798244	25
	3	64, 119, 176	15.820902860	342
	4	48, 88, 128, 181	18.655733570	11461
	5	48, 88, 128, 174, 202	21.401608305	469862

Goldhill	2	90, 157	12.546393623	24
	3	78, 131, 177	15.607747002	329
	4	65, 105, 147, 189	18.414213765	11958
	5	59, 95, 131, 165, 199	21.099138996	399458

Lake	2	91, 163	12.520359742	24
	3	72, 119, 169	15.566286745	336
	4	70, 111, 155, 194	18.365636309	12658
	5	64, 99, 133, 167, 199	21.024982760	410753

Aerial	2	68, 159	12.538208248	29
	3	68, 130, 186	15.751881495	347
	4	68, 117, 159, 200	18.615899102	11390
	5	68, 108, 141, 174, 207	21.210455499	599570

**Table 2:** Thresholds, objective function values, and time processing provided by the exhaustive search for Otsu's method.

Images	*K*	Threshold values	Objective function	Time (ms)
Barbara	2	82, 147	2608.610778507	39
	3	75, 127, 176	2785.163280467	89
	4	66, 106, 142, 182	2856.262131671	3014
	5	57, 88, 118, 148, 184	2890.976609405	100079

Living room	2	87, 145	1627.909172752	39
	3	76, 123, 163	1760.103018395	88
	4	56, 97, 132, 168	1828.864376614	2945
	5	49, 88, 120, 146, 178	1871.990616316	130397

Boats	2	93, 155	1863.346730649	38
	3	73, 126, 167	1994.536306242	89
	4	65, 114, 147, 179	2059.866280428	2931
	5	51, 90, 126, 152, 183	2092.775965336	75879

Goldhill	2	94, 161	2069.510202452	38
	3	83, 126, 179	2220.372641501	88
	4	69, 102, 138, 186	2295.380469158	2775
	5	63, 91, 117, 147, 191	2331.156597921	74674

Lake	2	85, 154	3974.738214185	39
	3	78, 140, 194	4112.631097687	89
	4	67, 110, 158, 198	4180.886161109	2613
	5	57, 88, 127, 166, 200	4216.943583790	73019

Aerial	2	125, 178	1808.171050536	46
	3	109, 147, 190	1905.410606582	103
	4	104, 134, 167, 202	1957.017965982	2670
	5	99, 123, 148, 175, 205	1980.656737348	99880

**Table 3:** Comparison of the mean values and standard deviations obtained for the PSO, DE, CS, FA, BA, and IBA based on Kapur's entropy criterion for six test images over 50 runs.

Alg.	*K*	Barbara		Living room		Boats		Goldhill		Lake		Aerial	
		Mean values	St. Dev.	Mean values	St. Dev.	Mean values	St. Dev.	Mean values	St. Dev.	Mean values	St. Dev.	Mean values	St. Dev.
PSO	2	12.668336540	5.33*E* − 15	12.405792709	1.64*E* − 04	12.574798244	1.42*E* − 14	12.546393623	7.11*E* − 15	12.520359742	5.33*E* − 15	12.538208248	1.78*E* − 15
	3	15.747087798	1.42*E* − 14	15.552015642	2.82*E* − 03	15.820679619	8.84*E* − 04	15.607747002	1.42*E* − 14	15.566286745	1.24*E* − 14	15.751881495	5.33*E* − 15
	4	18.549612938	1.94*E* − 02	18.467328310	6.64*E* − 03	18.640100415	3.00*E* − 02	18.414173744	2.07*E* − 04	18.357505953	2.02*E* − 02	18.615899102	1.78*E* − 14
	5	21.241857967	6.71*E* − 03	21.131564234	2.18*E* − 02	21.392020144	4.12*E* − 02	21.099092699	1.28*E* − 04	21.015922726	4.40*E* − 02	21.192396874	5.44*E* − 02

DE	2	12.668336540	5.33*E* − 15	12.405985592	5.33*E* − 15	12.574798244	1.42*E* − 14	12.546393623	7.11*E* − 15	12.520359742	5.33*E* − 15	12.538208248	1.78*E* − 15
	3	15.747087798	1.42*E* − 14	15.552578874	3.03*E* − 04	15.820902860	8.88*E* − 15	15.607743578	2.40*E* − 05	15.566286745	1.24*E* − 14	15.751881495	5.33*E* − 15
	4	18.556749938	1.51*E* − 04	18.470970822	3.16*E* − 04	18.655660844	1.64*E* − 04	18.603017275	1.78*E* − 03	18.365579671	2.79*E* − 04	18.615769177	6.36*E* − 04
	5	21.245566656	2.34*E* − 04	21.149062508	1.78*E* − 03	21.401458219	3.21*E* − 04	21.409946039	3.55*E* − 15	21.024780111	4.59*E* − 04	21.210411012	1.12*E* − 04

CS	2	12.668336540	5.33*E* − 15	12.405985592	5.33*E* − 15	12.574798244	1.42*E* − 14	12.546393623	7.11*E* − 15	12.520359742	5.33*E* − 15	12.538208248	1.78*E* − 15
	3	15.747087798	1.42*E* − 14	15.552622213	1.07*E* − 14	15.820902860	8.88*E* − 15	15.607747002	1.42*E* − 14	15.566286745	1.24*E* − 14	15.751881495	5.33*E* − 15
	4	18.556786861	2.49*E* − 14	18.471055578	2.49*E* − 14	18.655733570	1.07*E* − 14	18.414197322	6.53*E* − 05	18.365636309	1.78*E* − 14	18.615899102	1.78*E* − 14
	5	21.245645311	1.42*E* − 14	21.149400604	1.64*E* − 03	21.401608305	7.11*E* − 15	21.099125539	6.59*E* − 05	21.024962923	5.95*E* − 05	21.210455499	1.78*E* − 15

FA	2	12.668336540	5.33*E* − 15	12.405985592	5.33*E* − 15	12.574798244	1.42*E* − 14	12.546393623	7.11*E* − 15	12.520359742	5.33*E* − 15	12.538208248	1.78*E* − 15
	3	15.747087798	1.42*E* − 14	15.552622213	1.07*E* − 14	15.820902860	8.88*E* − 15	15.607747002	1.42*E* − 14	15.566286745	1.24*E* − 14	15.751881495	5.33*E* − 15
	4	18.556786861	2.49*E* − 14	18.471014902	2.85*E* − 04	18.655723798	4.79*E* − 05	18.414213765	2.13*E* − 14	18.365636309	1.78*E* − 14	18.615899102	1.78*E* − 14
	5	21.245645311	1.42*E* − 14	21.149483979	1.46*E* − 03	21.401583877	7.33*E* − 05	21.099138996	0.00*E* − 00	21.024982760	0.00*E* − 00	21.210455499	1.78*E* − 15

BA	2	12.668336540	5.33*E* − 15	12.405885825	1.52*E* − 04	12.574798244	1.42*E* − 14	12.546393623	7.11*E* − 15	12.520359742	5.33*E* − 15	12.538208248	1.78*E* − 15
	3	15.747087798	1.42*E* − 14	15.552622213	1.07*E* − 14	15.820902860	8.88*E* − 15	15.607747002	1.42*E* − 14	15.566286745	1.24*E* − 14	15.751881495	5.33*E* − 15
	4	18.555593147	8.36*E* − 03	18.469796068	3.52*E* − 03	18.644796771	2.68*E* − 02	18.414213765	2.13*E* − 14	18.356346829	2.13*E* − 02	18.615899102	1.78*E* − 14
	5	21.245645311	1.42*E* − 14	21.133082528	1.59*E* − 02	21.399205241	7.12*E* − 03	21.099138996	0.00*E* − 00	21.024982760	0.00*E* − 00	21.210452617	2.02*E* − 05

IBA	2	12.668336540	5.33*E* − 15	12.405985592	5.33*E* − 15	12.574798244	1.42*E* − 14	12.546393623	7.11*E* − 15	12.520359742	5.33*E* − 15	12.538208248	1.78*E* − 15
	3	15.747087798	1.42*E* − 14	15.552622213	1.07*E* − 14	15.820902860	8.88*E* − 15	15.607747002	1.42*E* − 14	15.566286745	1.24*E* − 14	15.751881495	5.33*E* − 15
	4	18.556786861	2.49*E* − 14	18.471055578	2.49*E* − 14	18.655733570	1.07*E* − 14	18.414213765	2.13*E* − 14	18.365636309	1.78*E* − 14	18.615899102	1.78*E* − 14
	5	21.245645311	1.42*E* − 14	21.150302316	1.78*E* − 14	21.401608305	7.11*E* − 15	21.099138996	0.00*E* − 00	21.024982760	0.00*E* − 00	21.210455499	1.78*E* − 14

**Table 4:** Comparison of the mean values and standard deviations obtained for the PSO, DE, CS, FA, BA, and IBA based on Otsu's criterion for six test images over 50 runs.

Alg.	*K*	Barbara		Living room		Boats		Goldhill		Lake		Aerial	
		Mean values	St. dev.	Mean values	St. dev.	Mean values	St. dev.	Mean values	St. dev.	Mean values	St. dev.	Mean values	St. dev.
PSO	2	2608.610778507	1.82*E* − 12	1627.909172752	0.00*E* − 00	1863.346730649	0.00*E* − 00	2069.510202452	4.55*E* − 13	3974.738214185	3.64*E* − 12	1808.171050536	2.27*E* − 13
	3	2785.163280467	2.27*E* − 12	1760.103018395	2.27*E* − 13	1994.536306242	1.59*E* − 12	2220.372641501	1.36*E* − 12	4112.631097687	4.55*E* − 12	1905.410606582	1.14*E* − 12
	4	2856.260804034	6.66*E* − 03	1828.864376614	1.59*E* − 12	2059.866220175	4.22*E* − 04	2295.380095430	1.48*E* − 03	4180.883976390	7.41*E* − 03	1955.085619462	7.65*E* + 00
	5	2890.975549258	5.05*E* − 02	1871.984827146	2.29*E* − 02	2092.771150715	8.36*E* − 03	2331.156479206	3.56*E* − 04	4216.942888298	3.99*E* − 03	1979.170306260	2.51*E* + 00

DE	2	2608.610778507	1.82*E* − 12	1627.909172752	0.00*E* − 00	1863.346730649	0.00*E* − 00	2069.510202452	4.55*E* − 13	3974.738214185	3.64*E* − 12	1808.171050536	2.27*E* − 13
	3	2785.162093432	8.31*E* − 03	1760.103018395	2.27*E* − 13	1994.535269293	7.26*E* − 03	2220.372641501	1.36*E* − 12	4112.631097687	4.55*E* − 12	1905.410606582	1.14*E* − 12
	4	2856.261305066	2.80*E* − 03	1828.860328016	1.30*E* − 02	2059.865271461	6.85*E* − 03	2295.380095430	1.48*E* − 03	4180.883976390	7.41*E* − 03	1955.085619462	7.65*E* + 00
	5	2890.971346990	2.05*E* − 02	1871.976701063	2.34*E* − 02	2092.766907541	2.71*E* − 02	2331.156479206	3.56*E* − 04	4216.942888298	3.99*E* − 03	1979.170306260	2.51*E* + 00

CS	2	2608.610778507	1.82*E* − 12	1627.909172752	0.00*E* − 00	1863.346730649	0.00*E* − 00	2069.510202452	4.55*E* − 13	3974.738214185	3.64*E* − 12	1808.171050536	2.27*E* − 13
	3	2785.163280467	2.27*E* − 12	1760.103018395	2.27*E* − 13	1994.536306242	1.59*E* − 12	2220.372641501	1.36*E* − 12	4112.631097687	4.55*E* − 12	1905.410606582	1.14*E* − 12
	4	2856.261511717	2.45*E* − 03	1828.864376614	1.59*E* − 12	2059.866280428	1.36*E* − 12	2295.380469158	2.27*E* − 12	4180.886161109	0.00*E* − 00	1957.017965982	0.00*E* − 00
	5	2890.976540127	4.85*E* − 04	1871.990230213	2.70*E* − 03	2092.775817560	1.03*E* − 03	2331.155240485	4.76*E* − 03	4216.943583790	9.09*E* − 13	1980.651043072	1.16*E* − 02

FA	2	2608.610778507	1.82*E* − 12	1627.909172752	0.00*E* − 00	1863.346730649	0.00*E* − 00	2069.510202452	4.55*E* − 13	3974.738214185	3.64*E* − 12	1808.171050536	2.27*E* − 13
	3	2785.163280467	2.27*E* − 12	1760.103018395	2.27*E* − 13	1994.536306242	1.59*E* − 12	2220.372641501	1.36*E* − 12	4112.631097687	4.55*E* − 12	1905.410606582	1.14*E* − 12
	4	2856.262131671	4.55*E* − 13	1828.864376614	1.59*E* − 12	2059.866280428	1.36*E* − 12	2295.380469158	2.27*E* − 12	4180.886161109	0.00*E* − 00	1957.017965982	0.00*E* − 00
	5	2890.976609405	3.64*E* − 12	1871.990616316	0.00*E* − 00	2092.773515829	3.57*E* − 03	2331.156597921	2.27*E* − 12	4216.943583790	9.09*E* − 13	1980.656737348	9.09*E* − 13

BA	2	2608.610778507	1.36*E* − 12	1627.909172752	2.27*E* − 13	1863.346730649	0.00*E* − 00	2069.510202452	4.55*E* − 13	3974.738214185	4.09*E* − 12	1808.171050536	2.27*E* − 13
	3	2785.163280467	1.36*E* − 12	1760.103018395	2.27*E* − 13	1994.536306242	1.14*E* − 12	2220.372641501	1.36*E* − 12	4112.631097687	3.64*E* − 12	1905.410606582	1.14*E* − 12
	4	2856.262131671	4.55*E* − 13	1828.864376614	2.27*E* − 12	2059.866280428	9.09*E* − 13	2295.380469158	2.27*E* − 12	4180.886161109	0.00*E* − 00	1957.017965982	2.27*E* − 13
	5	2890.976609405	2.73*E* − 12	1871.990616316	0.00*E* − 00	2092.772750357	3.78*E* − 03	2331.156597921	2.27*E* − 12	4216.943583790	3.64*E* − 12	1979.513584665	2.29*E* + 00

IBA	2	2608.610778507	1.36*E* − 12	1627.909172752	0.00*E* − 00	1863.346730649	0.00*E* − 00	2069.510202452	4.55*E* − 13	3974.738214185	4.09*E* − 12	1808.171050536	2.27*E* − 13
	3	2785.163280467	1.36*E* − 12	1760.103018395	2.27*E* − 13	1994.536306242	1.14*E* − 12	2220.372641501	1.36*E* − 12	4112.631097687	3.64*E* − 12	1905.410606582	1.14*E* − 12
	4	2856.262131671	4.55*E* − 13	1828.864376614	2.27*E* − 12	2059.866280428	9.09*E* − 13	2295.380469158	2.27*E* − 12	4180.886161109	0.00*E* − 00	1957.017965982	0.00*E* − 00
	5	2890.976609405	2.73*E* − 12	1871.990616316	0.00*E* − 00	2092.775965336	1.36*E* − 12	2331.156597921	2.27*E* − 12	4216.943583790	3.64*E* − 12	1980.656737348	9.09*E* − 13

**Table 5:** Mean of the CPU times (in milliseconds) and mean of the iteration numbers obtained for the PSO, DE, CS, FA, BA, and IBA based on Kapur's entropy criterion for six test images over 50 runs.

Alg.	*K*	Barbara		Living room		Boats		Goldhill		Lake		Aerial	
		Mean time (ms)	Mean iteration	Mean time (ms)	Mean iteration	Mean time (ms)	Mean iteration	Mean time (ms)	Mean iteration	Mean time (ms)	Mean iteration	Mean time (ms)	Mean iteration
PSO	2	2.18	9.22	102.82	1165.14	3.08	11.84	2.54	8.84	2.41	8.86	2.62	10.7
	3	3.30	14.28	21.96	218.56	16.23	136.58	3.18	13.54	3.65	14.58	3.24	13.9
	4	49.00	495.36	77.23	853.62	123.62	1367.86	10.22	97.86	26.99	295.76	4.03	19.76
	5	88.16	1050.8	153.53	1725.22	74.46	814.22	23.56	258.5	77.64	893.98	58.54	695.92

DE	2	3.56	14.59	4.34	18.92	3.38	16.92	2.15	16.44	5.0	15.74	3.81	17.01
	3	6.3	30.0	8.02	70.60	8.84	43.32	7.94	69.30	6.24	30.04	6.51	30.96
	4	24.46	240.68	33.62	322.91	46.92	477.16	48.36	125.48	22.12	165.08	14.12	124.56
	5	47.8	527.96	77.14	801.21	65.48	683.29	22.2	116.28	55.9	603.01	56.38	624.16

CS	2	71.04	194.54	53.30	129.84	53.84	135.58	82.29	209.48	70.48	183.36	56.32	150.88
	3	150.71	420.42	125.48	322.42	128.92	330.22	149.68	441.64	138.23	375.66	104.75	292.22
	4	189.31	518.58	222.48	570.6	170.28	436.02	180.31	500.11	220.44	604.58	174.84	482.26
	5	301.65	786.64	499.42	1303.8	247.15	631.36	280.55	720.34	336.29	915.92	193.21	532.76

FA	2	15.01	11.96	17.02	11.9	16.39	12.32	13.66	10.08	15.92	12.32	13.86	11.0
	3	34.07	29.82	37.24	29.7	36.24	29.24	32.08	28.66	34.56	29.42	32.83	29.18
	4	43.30	38.6	50.25	77.9	54.68	117.8	41.11	36.6	43.84	37.66	43.18	38.96
	5	50.15	44.04	104.74	515.06	76.22	241.94	48.95	42.01	50.31	43.6	50.46	45.46

BA	2	1.78	2.04	96.82	722.92	24.17	142.96	2	1.8	1.6	1.74	1.22	5.06
	3	2.52	6.74	89.78	734.8	64.96	421.48	2.84	6.94	2.32	8.82	3.6	14.4
	4	40.2	146.46	124.16	1098.72	115.34	969.66	6.76	37.18	56.88	397.8	32.6	134.52
	5	60.9	412.92	175.32	1718.82	98.42	785.04	30.92	157.92	39.24	238.12	64.48	463.78

IBA	2	2.12	9.14	6.34	25.14	5.36	10.02	2.88	8.92	2.28	9.18	2.7	11.18
	3	3.84	16.8	5.3	22.8	5.5	22.4	3.84	16.62	5	17.5	5.96	19.88
	4	7.16	26.26	9.92	35.48	10.3	43.3	6.28	28.82	7.14	26.98	7.48	30.98
	5	8.66	40.06	24.7	134.38	14.4	50.9	7.88	38.62	9.5	42.7	9.5	44.98

**Table 6:** Mean of the CPU times (in milliseconds) and mean of the iteration numbers obtained for the PSO, DE, CS, FA, BA, and IBA based on Otsu's entropy criterion for six test images over 50 runs.

Alg.	*K*	Barbara		Living room		Boats		Goldhill		Lake		Aerial	
		Mean time (ms)	Mean iteration	Mean time (ms)	Mean iteration	Mean time (ms)	Mean iteration	Mean time (ms)	Mean iteration	Mean time (ms)	Mean iteration	Mean time (ms)	Mean iteration
PSO	2	0.84	9.4	0.90	9.6	0.94	8.8	0.94	8.98	0.91	10.12	0.81	9.04
	3	1.22	13.26	1.24	14.68	1.28	14.1	2.86	14.3	1.17	13.48	1.29	15.16
	4	4.84	138.52	1.60	19.18	3.12	56.56	5.16	136.32	6.55	175.58	5.18	138.44
	5	10.02	261.06	8.14	221.64	32.96	1012.3	7.52	222.92	7.01	179.38	20.21	622.38

DE	2	0.73	14.06	1.0	15.66	1.0	16.59	0.81	15.14	0.76	17.72	0.85	15.9
	3	3.01	66.3	2.25	30.3	2.81	70.01	1.51	27.88	1.90	29.19	3.20	70.1
	4	8.0	199.22	11.12	282.01	4.72	120.58	7.60	199.5	7.42	200.23	7.01	162.5
	5	26.6	796.88	24.3	683.11	31.90	950.33	23.9	686.32	21.65	604.76	18.52	523.22

CS	2	35.80	179.0	30.03	223.34	32.74	204.04	27.88	195.30	38.04	267.66	32.45	254.10
	3	51.66	370.88	56.67	371.50	53.74	378.40	59.98	424.62	49.68	381.36	52.78	395.44
	4	100.78	723.22	83.43	578.94	76.98	595.66	70.92	531.74	85.30	646.44	83.84	593.52
	5	114.63	802.80	122.78	836.32	102.84	677.04	159.69	1115.44	109.90	746.36	209.36	1487.06

FA	2	8.16	12.02	8.72	12.54	7.17	10.64	8.61	12.68	7.28	11.66	7.94	12.18
	3	15.73	28.62	12.67	28.54	16.10	28.7	15.61	27.78	13.38	30.04	15.56	27.7
	4	17.84	37.7	19.16	38.78	18.43	38.2	17.12	37.66	18.49	38.86	18.05	38.58
	5	21.27	43.9	20.15	43.78	43.51	669.52	20.93	44.54	21.27	43.12	20.58	44.68

BA	2	0.4	1.86	0.4	1.66	0.42	1.58	0.42	1.75	0.38	1.7	0.38	1.64
	3	0.98	6.08	1.48	6.82	1.24	8.54	0.8	5.58	0.82	6.14	1.08	6.94
	4	3.68	30.16	3.56	28.72	4	33.56	3.72	30.36	4.48	31.32	5.1	42.14
	5	21.16	30.16	14.32	134.7	73.44	963.32	16.88	170.42	16.54	156.36	55.12	654.72

IBA	2	1.34	9.02	1.31	8.5	1.68	9.18	1.26	8.88	1.36	8.98	1.34	8.86
	3	2.38	16.36	2.32	16.44	2.24	16.34	2.44	16.6	2.36	16.54	2.36	16.38
	4	3.74	26.60	3.6	26.48	3.66	26.56	3.7	26.3	3.58	26.56	3.46	25.84
	5	6.88	38.62	5.3	39.2	7.16	52.48	6.38	40.14	5.36	37.48	5.66	41.08

**Table 7:** The percent of the best results for thresholds 4 and 5.

Alg.	Kapur's method	Otsu's method
PSO	8%	8%
DE	0%	0%
CS	67%	58%
FA	67%	92%
BA	42%	83%
IBA	100%	100%

**Table 8:** The number of evaluations for all test images and all threshold values for Kapur's method.

Alg.	Trsh. 2	Trsh. 3	Trsh. 4	Trsh. 5	Total
PSO	1214	411	3130	5439	10194
DE	96	186	1456	3356	5094
CS	1004	2183	3112	4891	11189
FA	70	176	347	932	1525
BA	876	1193	2784	3777	8631
IBA	74	116	192	352	734

**Table 9:** The number of evaluations for all test images and all threshold values for Otsu's method.

Alg.	Trsh. 2	Trsh. 3	Trsh. 4	Trsh. 5	Total
PSO	56	85	665	25206	3326
DE	95	294	1164	4245	5798
CS	1323	2322	3669	5665	12979
FA	72	171	230	889	1362
BA	10	40	196	2110	2356
IBA	53	99	158	249	559
